# National Society of Surgery

**Published:** 1860-03

**Authors:** 


National Society of Surgery.—We have noticed that the "Society of Surgery, of Paris," established many years since, has recently been taken under the patronage of the French government, as an association of "public utility."

The society at its origin, had to contend with some difficulties owing to prejudices and jealousies; but it has fairly earned its reputation, and may now be regarded in the light of successor to the old French Academy of Surgery, whose action exerted so great and favorable an influence on the progress of the art, and whose "memoirs" are still so often quoted.

In view of the success of the "Society of Surgery, of Paris," the thought naturally occurs, why would not a similar association, composed of hospital surgeons, professors, and others interested in this branch, be advantageous in this country. It might meet like the physicians of the lunatic asylums, quietly, confine its labors to matters of a purely scientific nature, and be made agreeable as well as useful to its members.

---

To Subscribers.—Correspondents and those sending us their names as subscribers for the *Journal*, will save us much trouble, and insure to themselves prompt attention to any requests contained in their letters, by giving their address in full. So many towns in different States have the same name, that when the name of the State is omitted, we are often entirely at a loss where to mail the *Journal*, or direct answers to letters.

Any subscribers who do not receive the *Journal* regularly, will confer a favor by giving us immediate notice of that fact and if the fault rests with us, it will at once be corrected.

---

State Medical Society.—The undersigned, the chairman of the Committee of Surgery respectfully requests any member of the profession having knowledge of interesting facts relating to surgical subjects, to communicate the same to him before May 1, 1860. D. BRAINARD.

---

☞ The annual meeting of the Illinois State Med. Society, for 1860, will take place at Paris, Edgar Co., on the second Tuesday in May.

